# Enhancement of Fracture Toughness of Epoxy Nanocomposites by Combining Nanotubes and Nanosheets as Fillers

**DOI:** 10.3390/ma10101179

**Published:** 2017-10-19

**Authors:** Nadiim Domun, Keith R. Paton, Homayoun Hadavinia, Toby Sainsbury, Tao Zhang, Hibaaq Mohamud

**Affiliations:** 1 School of Engineering, Kingston University, London, SW15 3DW, UK; n.domun@kingston.ac.uk (N.D.); t.zhang@kingston.ac.uk (T.Z.); 2 National Physical Laboratory, Hampton Road, Teddington, Middlesex, UK; toby.sainsbury@gmail.com (T.S.); hibaaq.mohamud@npl.co.uk (H.M.)

**Keywords:** epoxy, carbon nanotube, boron nitride nanotube, graphene, boron nitride nanosheet, hybrid nanocomposite, fracture toughness

## Abstract

In this work the fracture toughness of epoxy resin has been improved through the addition of low loading of single part and hybrid nanofiller materials. Functionalised multi-walled carbon nanotubes (f-MWCNTs) was used as single filler, increased the critical strain energy release rate, G_IC_, by 57% compared to the neat epoxy, at only 0.1 wt% filler content. Importantly, no degradation in the tensile or thermal properties of the nanocomposite was observed compared to the neat epoxy. When two-dimensional boron nitride nanosheets (BNNS) were added along with the one-dimensional f-MWCNTs, the fracture toughness increased further to 71.6% higher than that of the neat epoxy. Interestingly, when functionalised graphene nanoplatelets (f-GNPs) and boron nitride nanotubes (BNNTs) were used as hybrid filler, the fracture toughness of neat epoxy is improved by 91.9%. In neither of these hybrid filler systems the tensile properties were degraded, but the thermal properties of the nanocomposites containing boron nitride materials deteriorated slightly.

## 1. Introduction

Epoxies with high modulus and strength are used as the main component for adhesives and matrices for many types of applications and across a wide range of sectors. They are used as matrices in fibre reinforced polymer (FRP) composite materials from massive advanced lightweight structures, such as Boeing 787 and Airbus A350 fuselage, and large wind turbine blades to small equipment such as tennis rackets [1,2,3]. Developed in 1960s, the diglycidyl ether of bisphenol A (DGEBA) resin system is the most commonly used epoxy. After the epoxy resin reacts with a suitable curative, three-dimensional cross-linked thermoset structures are obtained which results in high modulus, high failure strength and improved interfacial bonding relative to other polymeric based adhesives. However, low fracture toughness is one of the key drawbacks of epoxies and many researchers have focused their works on increasing the fracture toughness of epoxy [4].

A common approach for improving the fracture toughness of epoxy is by addition of filler(s) to the epoxy matrix, introducing new mechanisms for energy absorption during the fracture processes [5,6]. Various filler materials have been used to achieve the enhancement of fracture toughness, including nanoclays [7], low-modulus rubber particles [8] and silica nanoparticles [6]. In recent years, the use of one-dimensional (1D) and two-dimensional (2D) nanomaterials as a reinforcing material component in polymer composites has attracted a significant amount of research attention. The remarkable strength and stiffness, coupled with very high aspect ratios of carbon nanotubes (CNTs) (up to 10^7^ for single-walled tubes [9]) make them a promising candidate as an effective reinforcing phase [10]. In addition the high electrical conductivity of CNTs allows for the simultaneous improvement in electrical conductivity and mechanical properties of the polymer. For applications where electrical conductivity needs to be avoided, boron nitride nanotubes (BNNTs) share many of the structural and mechanical properties, but possess a large band-gap, and so remain insulating. Ulus et al. studied the effect of boron nitride nanotubes on the mechanical properties of epoxy based nanocomposites [11], finding an increase of 25% in UTS was achieved at 0.5 wt% loading in comparison with the neat epoxy. Beyond this loading, the UTS degraded with increasing the loading content.

Although the 1D reinforcing materials have provided impressive improvements in the mechanical properties, the production costs generally remain high, limiting the commercial use of these materials. Following the isolation of monolayer graphene in 2005 and subsequent developments in production methods, graphene and few-layer graphene (FLG) has been intensively investigated as a reinforcing material. For example, Shokrieh et al. studied the effects of graphene nanoplatelets (GPLs) and graphene nanosheets (GNSs) on the fracture toughness of an epoxy [12] and they reported the fracture toughness of the epoxy nanocomposites increased by 39% and 16% respectively when GPLs and GNSs were incorporated at 0.5 wt% loading. The inelastic matrix deformation and nucleation of voids were the main cause for such enhancement.

Similar to CNTs nanomaterial, the isostructural hexagonal boron nitride has also been identified as potential filler, imparting similar mechanical properties, but without increasing the electrical conductivity. Lee et al. investigated the influence of surface modifications on the mechanical properties of epoxy-hexagonal boron nitride nanoflake (BNNF) nanocomposites [13]. The inclusion of 0.3 wt% of BNNF in epoxy provided an increase in the stiffness, UTS and toughness of the nanocomposites of 21%, 53% and 107%, respectively. They concluded that the surface functionalisation through 1-pyrenebutyric (PBA) enabled the PBA molecules to interact with the BNNFs and prevent agglomeration of BNNFs resulting in improvement in contact surface area between the nanofiller and the matrix system.

While the use of 1D and 2D materials as reinforcing fillers in polymer composites can provide improvements in mechanical properties, a 3-phase composite, combining 1D and 2D nanomaterials has recently been investigated [14,15,16]. For example, Yang et al. [14] studied the synergetic effects of graphene platelets and carbon nanotubes on the mechanical and thermal properties of epoxy composites. The introduction of both nanofillers in epoxy resulted in higher ultimate tensile strength (UTS), an increase of 35.4% over the neat epoxy in comparison with only 0.9% increase in UTS when only graphene nanoplatelets were dispersed into the epoxy [14]. However, the strong van der Waals forces between the fillers results in uneven dispersion of nanofillers and consequently, the composite properties fall short of the expectations associated with the promise of individually dispersed nanofillers [17]. A synergistic effect for enhancement of the mechanical and electric conductivity properties of epoxy composites was also demonstrated using three fillers, GNPs, carbon black (CB) and CNTs [18]. It was shown that the synergy originated not only from the dispersion promotion of fillers, but also from the effective link of both the narrow and broad gaps between graphite sheets by the spherical CB and long flexible CNTs, resulting in the formation of excellent conducting network in the matrix.

We have previously shown that the fracture toughness of epoxy can be improved by over 50% by the addition of 0.25 wt% functionalised graphene nanoplatelets (f-GNP) [19]. Importantly, there was no degradation in tensile or thermal properties of the resulting composites. In this paper we extend further our previous work by investigating the effect on mechanical properties of the epoxy by the synergetic effect of adding a combination of 1D and 2D nanofillers to the epoxy. The effects of nitric acid treated multi-walled carbon nanotubes (f-MWCNTs) on the tensile, fracture toughness and thermal properties of the resulting epoxy nanocomposites were systematically studied. Next hybrid 1D/2D nanofiller systems with either f-GNP/BNNT or BNNS/f-MWCNTs were also studied to investigate their effects on the tensile, fracture toughness and thermal properties of epoxy nanocomposites.

## 2. Results

### 2.1. f-MWCNT/Epoxy Nanocomposite

#### 2.1.1. Characterisation of f-MWCNT

**Figure 1:**
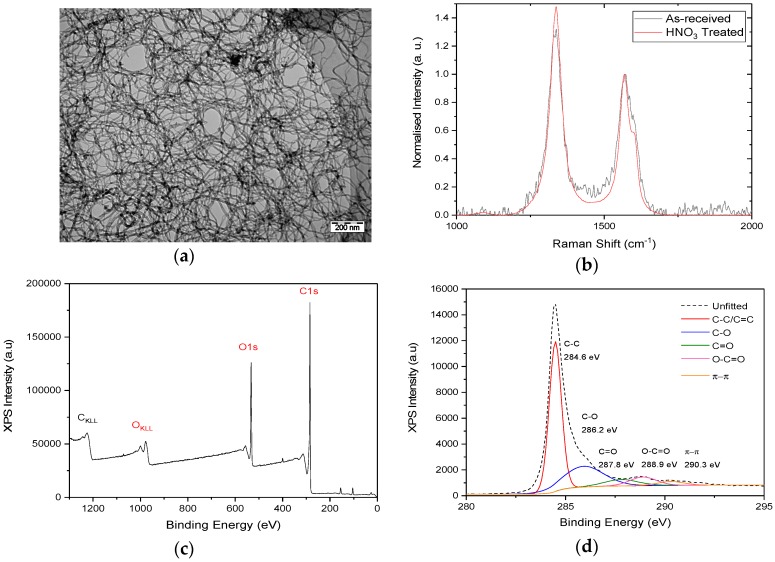
Characterisation of f-MWCNTs. (**a**) TEM of f-MWCNTs; (**b**) Raman spectra of as-received and functionalised MWCNTs; (**c**) Survey XPS scan of f-MWCNTs; (**d**) XPS spectrum of C1s region from f-MWCNTs.

Scanning electron microscopy of the f-MWCNTs, shown in Figure 1a, shows entangled tubes with an external diameter of 22 ± 6 nm, slightly larger than that reported by the supplier. Due to the entangled nature of the tubes, the lengths cannot be measured, but using the reported average length of 1.5 μm, the average aspect ratio of the tubes is between 53 and 94. The Raman spectrum of the HNO_3_-treated MWCNTs (Figure 1b) shows a downshift of around 4 cm^−1^ in the position of both the D-peak and G-peak, relative to the as-received material. The intensity ratio I_D_/I_G_ also increases from 1.22 to 1.54 following HNO_3_ treatment. At low functionalisation levels, this ratio is closely related to level of disorder in graphite materials [20,21], confirming that the acid treatment used here has introduced chemical functionalisation to the MWCNTs. This is supported by the XPS measurements (Figure 1c,d), which shows a significant oxygen signal, corresponding to a C/O ratio of 63.7. The C1s spectrum of the f-MWCNTs shows significant signals from C–O, C=O and O–C=O bonding, consistent with carbonyl, carboxyl and epoxide groups added to the walls of the nanotubes.

#### 2.1.2. Tensile Properties of f-MWCNT Nanocomposite

**Figure 2:**
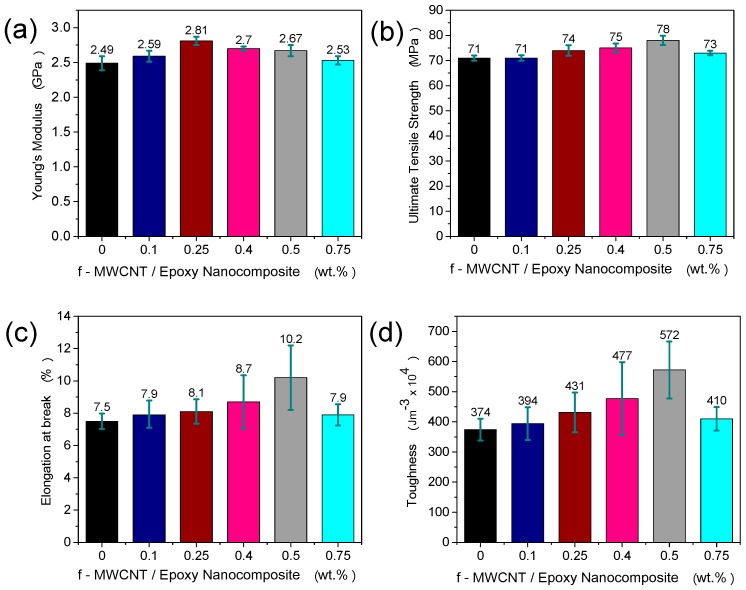
(**a**) Young’s modulus, (**b**) ultimate tensile strength (UTS), (**c**) elongation at break (%), and (**d**) toughness obtained from stress-strain curve of neat epoxy and f-MWCNT/Epoxy nanocomposites. In all cases, error bars indicate standard deviation.

The effect of loading of the f-MWCNT on the properties of the epoxy resins was studied using tensile test. The stiffness, strength, ductility and toughness of the nanocomposites as a function of f-MWCNT loading are shown in Figure 2. The values plotted are the means of the six specimens tested, and the error bars show the standard deviation calculated for each nanofiller loading. The average values and percentage of increase over the neat epoxy matrix are also shown in Table 1.

The Young’s modulus (E) of the f-MWCNT/epoxy nanocomposite shows an increase of 12% at 0.25 wt% loading (2.81 ± 0.1 GPa) before declining to close to the value of the control at 0.75 wt%. In contrast, no significant increase was found in the UTS of the nanocomposites until a loading of 0.5 wt%. At this loading the UTS was 78 ± 2 MPa, a 9.8% increase over the control neat epoxy. Increasing the loading further to 0.75 wt% led to a decrease in the UTS, again, not significant improvement over the pure epoxy.

This decrease in properties can be explained as the result of non-optimised dispersion of the filler material at these higher loadings. As the nanofiller loading increases, more energy is required during sonication to fully disperse the nanoparticles in the matrix. However, this also increases the risk of damaging the nanotube structure and the reduction in the effective length of the f-MWCNT. For this particular study, the processing of each loading contents was carried out using the same dispersion parameters across the full range of filler loading, and so the mixing process may not be fully optimised for the higher loadings.

Similar trends were observed for both elongation at break (EL) and toughness measured from the area under the stress-strain curve (T). At a loading level of 0.5 wt%, increases in elongation at break (EL) and toughness of 36% and 53% respectively were found over the pure epoxy control sample. As seen for the modulus and UTS, further increase in filler content leads to degradation in the ductility and toughness, in both cases, returning close to the neat epoxy control sample. Again, the deterioration in these properties at high loading content can be linked to the agglomeration of the MWCNTs which can lead to defect in the matrix, causing premature failure. We note however that there is a large spread in the values of EL and T measured here, which is illustrated by the error bars. Further testing would therefore be required to validate these results as genuine improvements based only on the mean values.

#### 2.1.3. Measuring Fracture Toughness of f-MWCNT Nanocomposite

**Figure 3:**
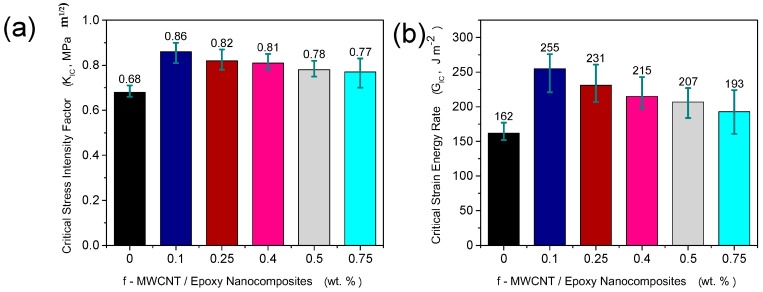
(**a**) Critical stress intensity factor, K_IC_, and (**b**) critical strain energy release rate, G_IC_, for f-MWCNT/Epoxy nanocomposites for different nanofiller loading. In all cases, error bars indicate standard deviation.

The effect of adding f-MWCNTs on the critical stress intensity factor, $K_{\mathrm{IC}}$, and the critical strain energy release rate, $G_{\mathrm{IC}}$, was studied using the single-edge-notch and 3-point bending (SENB) tests. As seen in Figure 3, and summarised in Table 2, a significant improvement in the fracture toughness was obtained at very low f-MWCNT loading. At loading of only 0.1 wt%, the critical strain energy release rate is increased by 57% over the pure epoxy. The value of K_IC_ is also increased by 26% at this loading. While this is a significant improvement in fracture toughness at very low filler content, further increases in fracture toughness are not seen at higher filler content. In fact, the fracture toughness falls as the filler loading is increased up to 0.75 wt%, at which point, it is not significantly different from the neat epoxy control sample. This may suggest that at higher loading of f-MWCNT stress concentrations due to re-agglomeration of f-MWCNT is occurring. This latter effect will reduce the stress transfer from the epoxy to the nanotubes, creating larger stress concentration areas. Similar behaviour from previous studies [22,23,24] carried out on nanocomposite reinforced with CNTs was reported, which caused reduction in the fracture toughness property of nanocomposites.

**Figure 4:**
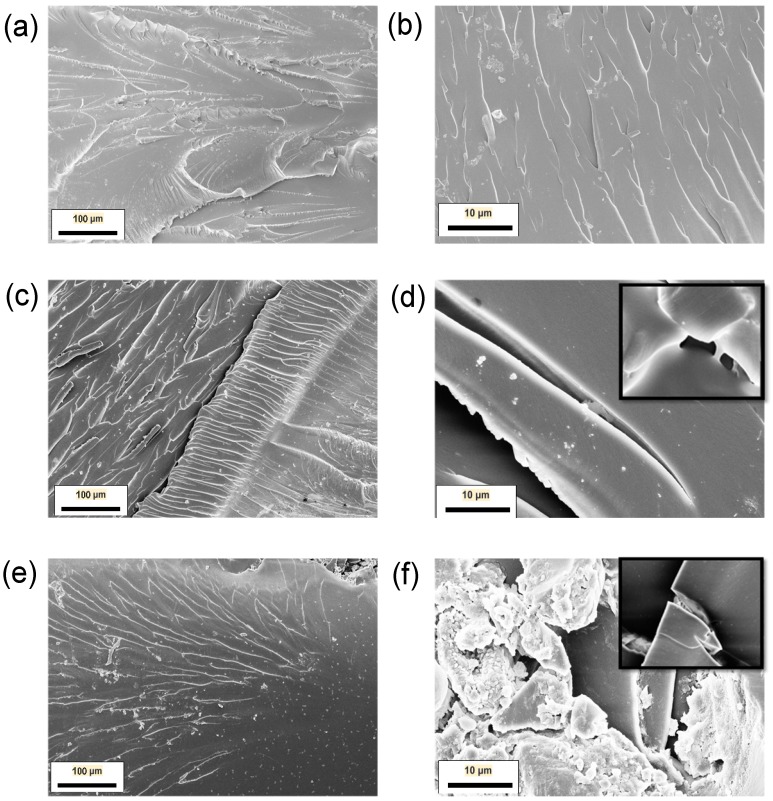
SEM micrographs of the fracture surface of neat epoxy at magnification (**a**) 500× and (**b**) 5000×; of 0.1 wt% f-MWCNT/EP at magnification (**c**) 500× and (**d**) 5000×; and of 0.75 wt% f-MWCNT/EP at magnification (**e**) 500× and (**f**) 5000×.

SEM analysis of the fracture surfaces has been carried out to investigate the toughening mechanisms in the nanocomposites (Figure 4). Overall, the samples reinforced with f-MWCNT show a rougher fracture surface than pure epoxy, as expected from increased toughness of the samples. The toughening mechanisms of polymers via the introduction of nanofillers have been widely investigated [25,26]. Micro-mechanical mechanisms that contribute to the toughening effect can be categorised as on-plane processes (crack pinning, crack deflection and immobilised layer of polymer) or off-plane processes (debonding) [27]. These mechanisms are often influenced by constraints such as the filler content, particle size and shape as well as chemical bonding between the nanofiller and the matrix system. Fracture surfaces of the unmodified epoxy (Figure 4a,b) show feather markings, which is indicative of crack forking in brittle failure.

In comparison, in the epoxy nanocomposite with 0.1 wt% of f-MWCNT, bowing lines (Figure 4) can be observed which is indicative of crack-pinning when the reinforcing particles are larger than the crack-opening displacement [12]. The occurrence of crack bridging which constitutes the f-MWCNT bundle to reduce the rate of crack propagation can also be observed in the inset of Figure 4d. The drop in fracture toughness property observed at higher loading content of 0.75 wt% is linked to the re-agglomeration of CNTs (see Figure 4f). These agglomerates of nano-filler can act as crack initiation sites, lowering the measured fracture toughness of the nanocomposite.

#### 2.1.4. Thermal Properties f-MWCNTs Nanocomposite

**Figure 5:**
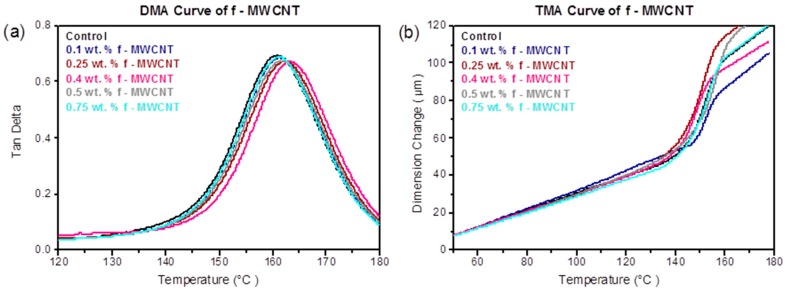
Damping behaviour (**a**) tan δ of f-MWCNT; (**b**) TMA curve of f-MWCNT.

Several aspects influence the nanocomposite’s T_g_, including the polymer structure, degree of cure, structural rigidity and changes in molecular weight due to addition of the nanofiller [28]. Dynamic Mechanical Analysis (DMA) was carried out on the neat epoxy and the nanocomposites made with different CNT loading to study nanofiller’s effect on the glass transition temperature, T_g_. The results show that there was only a slight increase in T_g_ across the whole range of the f-MWCNTs modified epoxy, confirming that the addition of the f-MWCNTs has not significantly compromised the usable temperature range of the epoxy (Figure 5a,b and Table 3). A maximum value of the glass transition temperature of 163 ± 0.4 °C was measured at 0.4 wt% f-MWCNT content, only 2 °C higher than neat epoxy at 161 ± 0.3 °C.

The coefficient of thermal expansion (CTE) was measured by TMA, with the values measured below T_g_, as shown in Table 3. As the content of f-MWCNTs increased, the value of CTE also increased, from (85 ± 0.3) × 10^−6^ K^−1^ for the pure epoxy, to (90 ± 1.2) × 10^−6^ K^−1^ at 0.25 wt%. Further increases in the f-MWCNT loading led to a fall in the CTE, with the value at 0.75 wt% only slightly higher than the pure epoxy. The increase in measured CTE by adding f-MWCNTs supports the suggestion of reduced cross-linking of the epoxy as discussed before.

The reinforcement effect of f-MWCNTs measured here can be compared with the results from our previous study [19] on f-GNP based epoxy at low loading content. In both cases, the tensile properties were maintained or showed only slight improvements, with slight increase in glass-transition temperature. While both filler materials also show an improvement of over 50% in fracture toughness, this is seen at lower filler content for f-MWCNTs that for f-GNPs (0.1 wt% CNT compared to 0.25 wt% GNP). Achieving effective reinforcement at lower filler content is advantageous in order to reduce costs, suggesting that f-MWCNTs may be a better choice than f-GNPs. Furthermore, the two-dimensional f-GNP has a higher risk of being agglomerated than the one-dimensional f-MWCNT due to its structural shape, larger interfacial areas as well as plane to plane contact areas.

### 2.2. Hybrid Nanofillers

A promising approach that has been extensively scrutinised in an attempt to further increase the properties of nanocomposites is the combination of 1D and 2D nanofillers. We have therefore investigated the hybridisation of 1D and 2D nanomaterials to explore whether higher mechanical and fracture toughness properties of the nanocomposites are achievable. As improvements in fracture toughness is the primary goal of the current work, we have used the filler content that yielded the maximum improvement in one-component nanofiller, i.e. 0.1 wt% for f-MWCNTs and 0.25 wt% for f-GNPs [19]. A low content (0.1 wt%) of boron–nitride nanotubes (BNNTs) or boron nitride nanosheets (BNNS) was then added to yield hybrid 1D/2D nanofillers and the tensile, fracture and thermal properties of the resultant epoxy nanocomposites have been obtained. Note that the total nano-filler content is therefore 0.2 wt% and 0.35 wt% for the f-MWCNT/BNNS and f-GNP/BNNT samples, respectively.

#### 2.2.1. Tensile Properties of Nanocomposites with Hybrid 1D/2D Nanofillers

**Figure 6:**
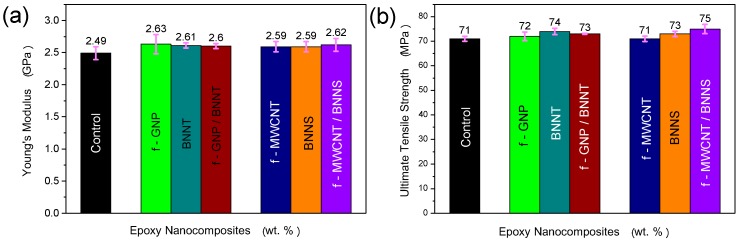

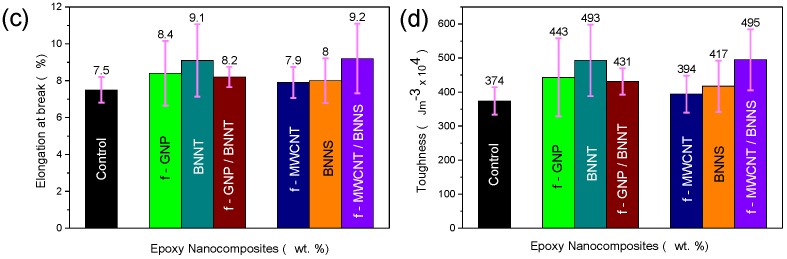
(**a**) Young’s modulus, (**b**) ultimate tensile strength (UTS), (**c**) elongation at break (%), and (**d**) toughness from stress-strain curve of neat epoxy, and epoxy with single component and the hybrid nanofiller systems. The filler content for each sample was: 0.25 wt% f-GNP; 0.1 wt% BNNT; 0.1 wt% f-MWCNT; 0.1 wt% BNNS. In all cases, error bars indicate standard deviation.

The tensile properties of the hybrid nanocomposites are measured and compared as shown in Figure 6 and the results are summarised in Table 4. There are no significant improvements in the measured modulus or UTS of the nanocomposites with two component hybrid nanofiller, over the neat epoxy control or the single component filler samples. While the ductility and toughness of the f-MWCNT/BNNS composite shows an improvement over the pure epoxy, the spread of results also increases, so it is not clear if this is a significant increase. Similarly, the hybrid f-MWCNT/BNNS epoxy nanocomposite has a higher toughness than the BNNS-only sample, but due to the spread of results, it is not clear if this is significant. Critically there is no degradation, as may have been expected, from higher total filler loading.

#### 2.2.2. Measuring Fracture Toughness of Nanocomposites with Hybrid 1D/2D Nanofillers

**Figure 7:**
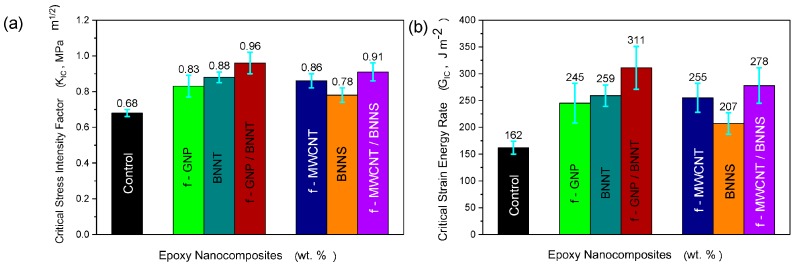
(**a**) Critical stress intensity factor, K_IC_, and (**b**) critical strain energy release rate, G_IC_, for the neat epoxy, and epoxy with single component and the hybrid nanofiller systems. In all cases, error bars indicate standard deviation. The filler content for each sample was: 0.25 wt% f-GNP; 0.1 wt% BNNT; 0.1 wt% f-MWCNT; 0.1 wt% BNNS.

In contrast to the tensile tests, mode I fracture toughness of the hybrid systems show significant improvements over the neat epoxy, as shown in Figure 7 and Table 5. For the f-GNP/BNNT system, the critical strain energy release rate of the combined composite was 92% higher than the pure epoxy control sample, and 27% higher than the f-GNP only sample. For the f-MWCNT/BNNS system, the improvement over the pure epoxy is only 71%, still very significant, but in this case, there is no significant increase over one component f-MWCNT filler nanocomposite. While these results are impressive, it should be noted that the filler content of the inorganic phase has not been optimised, and as such, further improvements may be possible. This is particularly true for the BNNTs, where removal of the non-tubular debris would be expected to yield further improvements.

The effect of using multiple fillers to modify the properties of epoxy matrix benefited from forming a co-supporting network as suggested by Kumar et al. [29]. The hybrid structure that is formed using the 1D and 2D nanomaterials shielded the fillers from potential fracture and damage during the processing stage. Moreover, the re-stacking of discrete 2D graphene nanoplatelets is efficiently inhibited by the introduction of the 1D BNNT. The presence of high contact area between the GNP and the BNNT due to the long BNNT bridging adjacent GNP is another explanation of the improvement in fracture toughness property of the nanocomposites. The synergy effects of the 1D and 2D nanofillers co-forming a 3-D hybrid structure is clearly contributing to such impressive enhancement [30].

**Figure 8:**
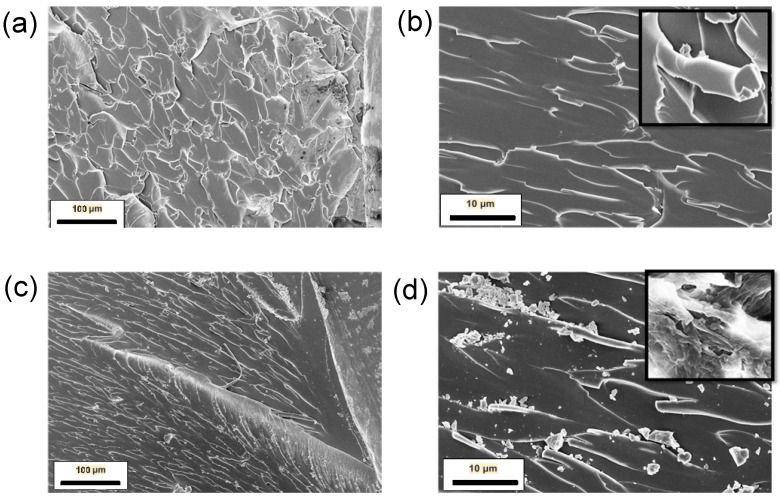
Fracture surface SEM images of the hybrid nanocomposites at magnification (**a**) 500× and (**b**) 5000× for 0.25:0.1 wt% (f-GNP:BNNT)/Epoxy; at magnification (**c**) 500× and (**d**) 5000× for 0.1:0.1 wt% (f-MWCNT: BNNS)/Epoxy.

SEM fractography analysis of the hybrid nanocomposite systems after the SENB test shows similar morphology to that seen in the f-MWCNT samples. Bowing lines are seen, suggesting that crack-pinning is still occurring, leading to the increase in fracture toughness. The coarse nature of the fracture surfaces observed in Figure 8a,b for both hybrid systems indicates that the inclusion of the 1D and 2D nanofillers have led to the deflection of crack path [31]. This development initiates off-plane loading that originate new fracture surfaces, hence leading to an increase in the mode I fracture toughness. There is no evidence that the toughening mechanisms are different when using combined 1D/2D reinforcement compared to using 1D or 2D alone.

#### 2.2.3. Thermal Properties of Nanocomposites with Hybrid 1D/2D Nanofillers

**Figure 9:**
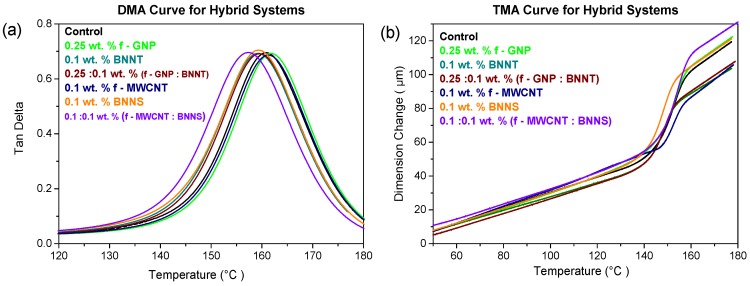
Damping behaviour (**a**) tan δ of hybrid systems and (**b**) TMA curve of hybrid systems.

While the fracture toughness has shown significant improvements, the thermal properties have been found to degrade slightly. As shown in Figure 9 and Table 6 there is a slight fall in T_g_ for the composites containing boron nitride, either as nanotubes or nanosheets. The lowest value was found for the f-MWCNT/BNNS composite, where the value of T_g_ was 157 ± 0.5°C around 4 °C below the neat epoxy. This decrease in T_g_ on addition of boron nitride contrasts with what has been previously reported by Yu et al. [32], who showed an increase in the T_g_ values at 5 wt% loading of boron nitride nanoplatelets. The fall in T_g_ seen in the current study may indicate that the chain mobility has increased on addition of boron nitride fillers. It is not clear why this would be the case, but may indicate that the processing needs to be optimised when using these materials.

The value of CTE for nanocomposites containing boron nitride filler materials is significantly higher than the samples containing only f-MWCNT (see Table 3). Boron nitride nanotubes result in a greater increase in CTE than the nanosheets, with the BNNT-only composite having a CTE of (110 ± 1.1) × 10^−6^ K^−1^. The higher values seen here suggest that the nanocomposites produced in this work may have incomplete curing of the epoxy as a result of the boron nitride additives. These higher values of CTE can limit the temperature range that these materials could be used at, and the materials that they can be combined with in manufactured components.

## 3. Materials and Methods

### 3.1. Materials

The resin used in this study was a two-part low viscosity epoxy, Araldite^®^ LY 564 resin and cycloaliphatic polyamine Aradur^®^ 2954 hardener purchased from Huntsman (Cambridge, UK). The normal ratio of resin to hardener was 100:35 by weight, with a gel time of approximately 90 min at 60 °C. Multi-walled carbon nanotubes (MWCNT) NC3100 were purchased from Nanocyl SA (Sambreville, Belgium) which were produced by catalytic chemical vapour deposition (CCVD) process. The average diameter of the MWCNT was given as ~9.5 nm with an average length of 1.5 μm and a carbon purity of >95.0%. The HNO_3_, methanol and ethanol used were of analytical grade and were obtained from Sigma-Aldrich (Poole, UK). Multi-Walled Boron Nitride Nanotubes (BNNT) purchased from NAiEEL Technology (Daejeon, South Korea) had an average diameter of 100 nm with length >1 μm and were used as-received. Hexagonal boron nitride powder (h-BN) was purchased from UK Abrasives, Inc (Northbrook, IL, USA).

The f-MWCNTs used in this study were prepared with the following procedure [33]. Unmodified MWCNTs (0.1 g) were dispersed in 100 ml of HNO_3_ (70%) in a round-bottom flask (250 mL) equipped with a condenser and refluxed at 135 °C for 24 h. Next, the resulting mixture was diluted in deionised (DI) water (18.2 MΩ·cm) and filtered on a Millipore™ Isopore filter membrane (Millipore, Watford, UK). The collected solid was then repeatedly washed with DI water, methanol, and ethanol, until a neutral pH was reached, and subsequently dried in vacuum at 40 °C.

Functionalized Boron Nitride Nanosheet (BNNS) was prepared by the thermal treatment of hexagonal boron nitride in air. In a typical experimental run, 20 g h-BN powder was placed in a quartz tube in a tube furnace. The furnace was heated to 1000 °C and held at that temperature for two hours in air, and then washed with hot water.

**Figure 10:**
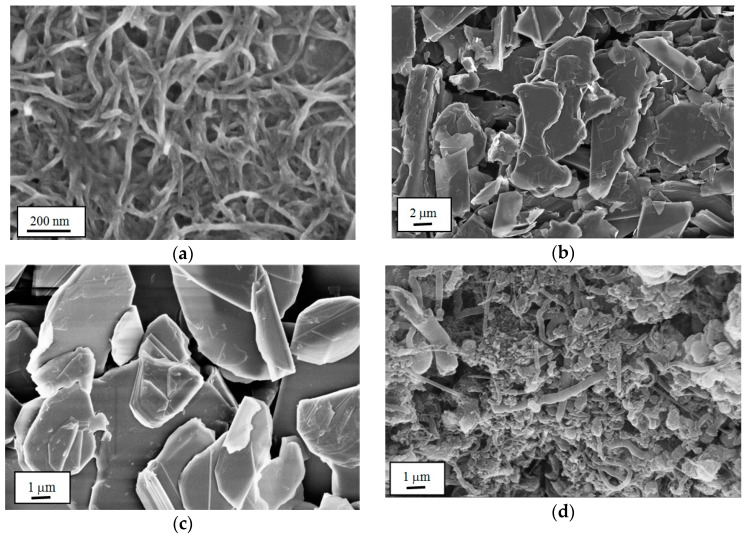
SEM images of nanofillers used in hybrid nanocomposites: (**a**) MWCNT; (**b**) f-GNP; (**c**) BNNS; (**d**) BNNTs.

Figure 10 shows SEM images of the 1D and 2D nanofillers used, MWCNT, f-GNP, BNNS and BNNT. The BNNTs were used as-received, which as shown in Figure 10d includes a significant portion of debris, comprising unreacted boron and iron catalyst. The concentration of tubes is therefore lower than that quoted here.

### 3.2. Characterization Methods

Scanning Electron Microscopy (SEM) was carried out using a ZEISS Supra (Zeiss, Oberkochen, Germany) at an accelerating voltage of 5 kV and a nominal working distance of 2.5 mm. The nanocomposite specimens were coated in a thin layer of gold (3 nm) to prevent charging. Raman spectra were recorded using a DXR high resolution Raman Microscope (Thermo Fisher Scientific, Waltham MA, USA) equipped with Ar laser (irradiation wavelength 532 nm). X-ray Photoelectron Spectroscopy (XPS) (Kratos Analytical Ltd, Manchester, UK) was performed using a Kratos Axis Ultra DLD system using an Al monochromated X-ray source operated at 15 kV, 5 mA emissions. Analysis conditions used were 160 eV pass energy, 1 eV steps, 0.2 s dwell per step and 2 sweeps. Samples for XPS were prepared by evaporation of MWCNT from solution onto Si-wafer substrates.

### 3.3. Compounding of Epoxy Nanocomposites

**Figure 11:**
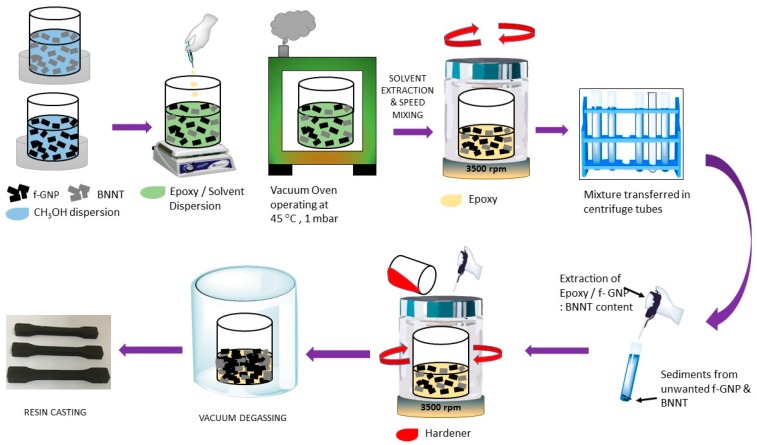
Schematic diagram of the processing of the f-GNP/BNNT based epoxy nanocomposite.

All of the nanocomposites in this study were prepared under identical conditions, as described previously from our previous study [19] and illustrated in Figure 11. Briefly, the filler material was dispersed by sonication in methanol, and then epoxy resin added dropwise, stirring continuously. The methanol was then removed in a rotorvap (45 °C, 10 mbar) then further held at 45 °C and 1 mbar for several days to ensure complete removal of the methanol. A high speed mixer (Speed Mixer DAC150 FVZ-K, Synergy Devices, High Wycombe, UK) operating at 3500 rpm for 12 min was then employed to mix the f-MWCNT/epoxy nanocomposite. The mixtures were left to stand for several days to allow un-exfoliated material to sediment, and the supernatant removed. This will result in a small, systematic, correction to the concentration of nanofillers in the final composites. The values reported here are therefore nominal concentrations.

Aradur^®^ 2954 hardener (Huntsman, Cambridge, UK) was then added to the resin/filler mixture, which was mixed again using a high speed mixer at 3500 rpm for 45 s. After degassing under vacuum for approximately 45 min, the filled epoxy was then poured into appropriate open aluminium moulds, casting dogbone specimens for mechanical; beam and cubic specimens for thermal testing. Specimens were cured for 1 h at 80 °C followed by 160 °C for 4 h. Samples were prepared with loadings between 0.1 wt% and 1 wt% in addition to a control sample of pure epoxy. Identical steps as described above were carried out for the hybrid nanocomposites consisting of two nanofillers, where each filler was co-dispersed in methanol during the initial phase of the processing as shown in Figure 11. The neat epoxy also underwent identical processing conditions as the nanofiller loadings during the processing stage.

### 3.4. Testing Procedure 

#### 3.4.1. Tensile Testing Procedure

**Figure 12:**
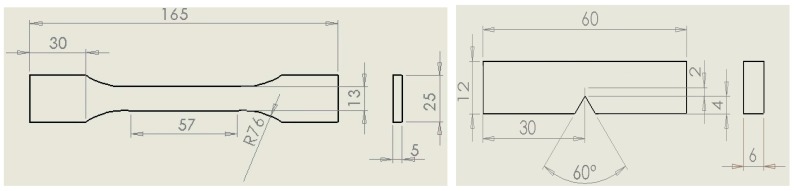
TYPE I tensile (**left**) and SENB (**right**) test specimen dimensions. All dimensions are in millimetres.

Uniaxial tensile tests on nanocomposites were performed on the cast dogbone specimens (see Figure 12) according to ASTM D638 (Type I geometry) [34] using an Instron^®^ universal test machine fitted with a 20 kN load cell. The crosshead speed was set at 2 mm/min and all tests were performed at room temperature. A clip-on extensometer with a 2.5 mm stroke was employed to measure the elongation during the test up to failure point. For each loading content six specimens were tested for statistical evaluation. The Young’s modulus (E), ultimate tensile strength (UTS), elongation at break (EL) and toughness (T) (the area under the stress-strain curve of the nanocomposites) were evaluated at the specific filler loadings.

In addition to the tensile toughness described above, the fracture toughness of the resulting nanocomposites was investigated according to ASTM D5045 [35] in three-point-bending using single-edge-notch bending (SENB) specimens as shown in Figure 12. A pre-crack of length 2 mm was introduced at the moulded V-notch by tapping a fresh razor blade. Tests were undertaken using an Instron^®^ test machine fitted with a 20 kN load cell at a crosshead speed of 10 mm/min. Six specimens were tested at each nanofiller loading content. The critical stress intensity factor, K_IC_, and the mode I fracture toughness, G_IC_, were calculated according to standard equations [35].

#### 3.4.2. Thermal Testing

A Q800 Dynamic Mechanical Analysis (DMA) was used to determine the T_g_ of the nanocomposites. The measurement point for T_g_ was taken from the peak of the tan δ curve. Rectangular specimens of dimensions 4 mm × 10 mm × 17.3 mm were clamped using a single cantilever clamp configuration with a torque of 1 N m. The measurements were undertaken in flexure at a fixed frequency of 1 Hz and a heating rate of 3 °C per minute. The temperature range used was from 40 °C to 200 °C. Three specimens were tested at each nanofiller loading content. Thermo-Mechanical Analysis (TMA) was performed using a TA Instruments Q400 to measure the dimensional changes in the nanocomposites as a function of temperature to obtain the CTE. A linear variable differential transformer (LVDT) was applied to each sample to detect any deformation due to expansion and contraction when subjected to a temperature profile. Measurements were carried out over the temperature range 40 °C to 180 °C at a heating rate of 3 °C per minute. Three specimens were tested at each nanofiller loading.

## 4. Conclusions

The mechanical, thermal and fracture toughness properties for a series of (1D) HNO_3_-treated MWCNTs (f-MWCNT)/epoxy nanocomposites at low loading content were investigated. Maximum UTS achieved at 0.5 wt% loading, a value of 78 MPa, compared to 71 MPa for the neat epoxy control system. An increment by 12.8% in stiffness occurred at 0.25 wt% loading for this particular nanocomposite series whilst the elongation at break experienced an increase of 36% in comparison with the neat epoxy control specimen. Optimal mode I fracture toughness, $G_{\mathrm{IC}}$, was found at 0.1 wt%, an impressive improvement of 57% higher than the neat epoxy.

Using the data acquired from this 1D and from our previous study on 2D plasma functionalised graphene nanoplatelets [19], we studied two hybrid nanofiller systems; one consists of functionalized multi-walled carbon nanotubes (f-MWCNT) with hexagonal boron nitride nanosheets (BNNS) and the other made of plasma functionalised graphene nanoplatelets (f-GNP) with boron nitride nanotubes (BNNT) in an attempt to further improve the fracture toughness performance of the nanocomposites. The synergy effect of mixing 1D and 2D nanofillers in epoxy matrix was thus explored. Combination of both f-MWCNT and BNNS at (0.1:0.1) wt% loading content resulted in a maximum UTS with an average growth of 5.6% relative to neat epoxy. For the instance of the hybrid f-MWCNT/BNNS epoxy nanocomposite, a growth of 71.6% was recorded for fracture toughness in comparison with the neat epoxy. Further to this improvement, a further increase in fracture toughness was attained at (0.25:0.1) wt% loading for the hybrid f-GNP/BNNT system, an impressive increment by 91.9% relative to the control system. Improvement in the tensile and fracture toughness properties of the nanocomposites with hybrid nanofillers can be explained due to the 1D nanofiller acting as prolonged arms with the 2D nanosheets, thereby exhibiting a 3D hybrid architecture. This interaction enables entanglement with the polymer chain achieving better interfacial bonding between the hybrid nanofillers and the epoxy resin. The toughening mechanisms were associated with crack bridging, crack pinning and deflection as observed from the fractography analysis. The glass transition temperature (T_g_) remained unchanged whilst the coefficient of thermal expansion (CTE) deteriorated for both of the hybrid systems.

## Figures and Tables

**Table 1:** Variation of E, UTS, % EL and T for different f-MWCNT loading relative to the neat epoxy.

CNT Loading wt%	E (GPa)	Increase (%)	UTS (MPa)	Increase (%)	EL (%)	Increase (%)	T $\left(Jm^{-3}\times 10^{4}\right)$	Increase (%)
Control	2.49	N/A	71	N/A	7.5	N/A	374	N/A
0.1	2.59	+4	71	0	7.9	+5	394	+5
0.25	2.81	+12	74	+4	8.1	+8	431	+15
0.4	2.70	+8	75	+5	8.7	+16	477	+27
0.5	2.67	+7	78	+9	10.2	+36	572	+52
0.75	2.53	+1	73	+2	7.9	+5	410	+9

**Table 2:** Variation of K_IC_ and G_IC_ for different f-MWCNT loading, along with percentage increase relative to the neat epoxy.

CNT Loading wt%	$K_{IC}\;\left(MPa\;m^{1∕2}\right)$	Increase (%)	$G_{IC}$ (J $m^{-2}$)	Increase (%)
Control	0.68	N/A	162	N/A
0.1	0.86	+26.4	255	+57.4
0.25	0.82	+20.6	231	+42.6
0.4	0.81	+19.1	215	+32.7
0.5	0.78	+14.7	207	+27.0
0.75	0.77	+13.2	193	+19.1

**Table 3:** Glass transition temperature T_g_ and CTE of the f-MWCNT series relative to the neat epoxy.

Loading of MWCNT (wt%)	Tg by DMA (°C)	CTE by TMA (×10^−6^ K^−1^)
Control	161 ± 0.3	85 ± 0.3
0.1	160 ± 0.1	89 ± 0.9
0.25	162 ± 0.3	90 ± 1.2
0.4	163 ± 0.4	87 ± 1.4
0.5	163 ± 2.2	88 ± 0.9
0.75	161 ± 0.9	87 ± 0.3

**Table 4:** Variation of E, UTS, %EL and T for epoxy with single component and the hybrid nanofiller systems relative to the control neat epoxy.

Nanofiller Loading (wt%)	E (GPa)	Increase (%)	UTS (MPa)	Increase (%)	EL (%)	Increase (%)	T $\left(Jm^{-3}\times 10^{4}\right)$	Increase (%)
Control	2.49	N/A	71	N/A	7.5	N/A	374	N/A
f-GNP (0.25)	2.63	+5.6	72	+1.4	8.4	+12.0	443	+18.4
BNNT (0.1)	2.61	+4.8	74	+4.2	9.1	+21.3	493	+31.8
f-GNP:BNNT (0.25:0.1)	2.6	+4.4	73	+2.8	8.2	+9.3	431	+15.2
f-MWCNT (0.1)	2.59	+4.0	71	0	7.9	+5.3	394	+ 5.3
BNNS (0.1)	2.59	+4.0	73	+2.8	8	+6.0	417	+11.5
f-MWCNT:BNNS (0.1:0.1)	2.62	+5.2	75	+5.6	9.2	+22.6	495	+32.4

**Table 5:** Variation of K_IC_ and G_IC_ for the hybrid nanocomposites relative to the neat epoxy.

Nanofiller (wt%)	$K_{IC}\;\left(MPa\;m^{1∕2}\right)$	Increase (%)	$G_{IC}$ (J $m^{-2}$)	Increase (%)
Control	0.68	N/A	162	N/A
f-GNP (0.25 wt%)	0.83	+22.1	245	+51.2
BNNT (0.1 wt%)	0.88	+29.4	259	+59.8
f-GNP:BNNT (0.25 wt%:0.1 wt%)	0.96	+41.2	311	+91.9
f-MWCNT (0.1 wt%)	0.86	+26.5	255	+57.4
BNNS (0.1 wt%)	0.78	+14.7	207	+27.0
f-MWCNT:BNNS (0.1 wt%:0.1 wt%)	0.91	+33.8	278	+71.6

**Table 6:** Glass transition temperature T_g_ and CTE of hybrid systems relative to the neat epoxy.

Loading (wt%)	Tg by DMA (°C)	CTE by TMA (×10^−6^ K^−1^)
Control	161 ± 0.3	85 ± 0.3
BNNT (0.1)	160 ± 0.3	110 ± 1.1
f-GNP:BNNT (0.25:0.1)	159 ± 0.9	104 ± 0.4
BNNS (0.1)	159 ± 0.5	93 ± 1.4
f-MWCNT:BNNS (0.1:0.1)	157 ± 0.5	92 ± 2.3
